# Foreign Honor Conferred on an American Surgeon

**Published:** 1852-11

**Authors:** 


*Foreign honor conferred on an American Surgeon.* — The honorary fellowship of King's and Queen's College of Physicians, in Ireland, has lately been conferred on Professor Valentine Mott. The honor of this distinction, measured by the number of persons on whom it has been conferred, is marked. It is stated that since the foundation of the college, nearly two centuries ago, but twenty-five medical men, of different countries, have received this degree — Prof. Mott being the only American.

